# The R3-MYB Transcription Factor DcMYB56 Regulates Anthocyanin Accumulation by Activating the Expression of Anthocyanin Biosynthesis-Related Genes in *Dendrobium candidum*

**DOI:** 10.3390/plants14121805

**Published:** 2025-06-12

**Authors:** Ning Jia, Wei Ye, Jinlan Jiang, Peiyu Wang, Jiqin Liu

**Affiliations:** 1 College of Agricultural and Forestry Science and Technology, Hebei North University, Zhangjiakou 075131, China; 2 Fujian Key Laboratory of Crop Genetic Improvement and Innovative Utilization for Mountain Area, Sanming Academy of Agricultural Science, Sanming 365509, China; yewei922@sina.com (W.Y.); fjsxjjl@163.com (J.J.); 17268144914@163.com (P.W.); 3 Institute of Plant Quarantine, Science and Technology Research Center of China Customs, Beijing 100026, China

**Keywords:** anthocyanin biosynthesis, DcMYB56, *Dendrobium candidum*, transcriptional regulation

## Abstract

*Dendrobium candidum* is a traditional Chinese medicinal herb with green and red stems. *D. candidum*, which has red stems, is highly nutritious. However, there is a need to clarify the mechanisms of transcriptional regulation underlying anthocyanin biosynthesis in *D. candidum*. In this study, we found that the red stem of *D. candidum* has a high anthocyanin content and a wide variety of types. Cyanidin derivatives of anthocyanins were found to be responsible for red pigmentation in red leaves. An R3-MYB transcription factor, DcMYB56, which modulates anthocyanin biosynthesis, along with homologs of other plants, was isolated and identified. Compared with that in green stems, DcMYB56 expression in *D. candidum* red stems markedly increased. The binding of DcMYB56 to the *DcCHS8*, *DcF3′H*, *DcF3′5′H*, and *DcANS1* gene promoters controlled DcMYB56 expression levels. The overexpression of DcMYB56 in *Arabidopsis thaliana* resulted in a red phenotype and substantially increased the anthocyanin content. Our findings suggest that DcMYB56 is important for anthocyanin biosynthesis, which thus sheds new light on the modulation of anthocyanin biosynthesis-associated transcription factors in *D. candidum*.

## 1. Introduction

The endangered epiphytic genus *Dendrobium* belongs to the family Orchidaceae, whose member species have applications in traditional Chinese medicine. The earliest records of this medicinal orchid were two thousand years ago in Shen Nong’s Herbal Classic, and it has a long history of therapeutic use [1]. Compared with other *Dendrobium* species, *D. candidum* is considerably rich in polysaccharides and alkaloids. Polysaccharides, bisphenols, phenols, alkaloids, amino acids, and trace elements are present in *D. candidum* [2]. It also has immunostimulant, antioxidant, antidiabetic, antitumor, and memory-enhancing properties [2,3,4,5,6,7,8]. The stems of *D. candidum* can have both green and red colors, with the latter indicating high-quality *D. candidum*, generally covering a larger planting area, and having greater market value than green *D. candidum*.

Anthocyanins are water-soluble flavonoid pigments, among which malvidin, cyanidin, pelargonidin, petunidin, delphinidin, and peonidin commonly occur in plant species [9] and confer red, blue, and purple colors [10]. Anthocyanins are the most important group of pigments in plants [11] and are partially responsible for pigment induction in flowers, fruits, and vegetables. The roles these compounds play in plants include protection against ultraviolet light and insect pests, attracting pollinators, and exhibiting antioxidant activity. Furthermore, they prevent atherosclerosis and some cancers, treat retinopathy, and improve vision [12]. The structural genes encoding key enzymes in the anthocyanin biosynthetic pathway have been extensively studied. Anthocyanin biosynthesis starts from the flavonoid biosynthetic pathway: Chalcone synthase (CHS) catalyzes CoA and malonyl CoA production to produce coumaroyl chalcone [13]. Then, chalcone isomerase (CHI), flavanone 3-hydroxylase (F3H), flavonoid 3′-hydroxylase (F3′H), flavonoid 3′,5′-hydroxylase (F3′5′H), dihydroflavonol 4-reductase (DFR), anthocyanin synthase (ANS), and UDP glucose: flavonoid-3-*O*-glucosyltransferase (UFGT) catalyze reactions to form stable anthocyanins [14]. Common anthocyanin aglycones include three basic anthocyanin aglycones, cyanidin, delphinidin, and pelargonidin, and their methylation products [15]. The genes encoding *CHS*, *DFR*, and *F3′5′H* in orchids have been identified [16]. A positive correlation between RrF3′H expression and the color intensity of *Rosa rugosa* flowers has been reported [17]. The F3′H mutation affects the seed coat and pubescence pigmentation of soybeans [18,19], suggesting a similar function of DcF3′H in *D. candidum*. However, F3′5′H serves as an important gene controlling anthocyanin biosynthesis [20], which is related to the formation of blue or purple pigments [21]. We detected increased *DcF3′5′H* expression and markedly increased delphinidin (Del), delphinidin 3-*O*-β-D-glucoside (Del-3-G), delphinidin 3,5-diglucoside (Del-3,5-D), and delphinidin-3-*O*-rutinoside chloride (Del-3-R) contents in *D. candidum* red stems compared with those in green stems, which may be related to red color formation [22]. Moreover, the *DcDFR1*, *DcANS*, and *DcUFGT* contents apparently increased in red *D. candidum*. MaDFR participates in flower color development in grape hyacinth. The anthocyanin accumulation and flower color intensity resulted from the differential MaDFR expression of *Nicotiana tabacum* [23]. Similarly, anthocyanin accumulation may be induced by BoDFR1 in pink-leaved ornamental kale [24], and anthocyanin biosynthesis in potato tubers may be promoted by *StANS* overexpression [25]. *UFGT* expression is positively related to anthocyanin content in grape varieties with different colors [26].

The interactions among structural and regulatory genes control anthocyanin biosynthesis. Transcription factors play important roles in modulating anthocyanin biosynthesis-related gene expression [27]. They were classified into various groups, such as R2R3-MYB, bHLH, and WD40 repeats, on the basis of their different DNA-binding domains [28]. These genes were widely expressed in numerous plants and are related to the regulation of anthocyanin biosynthesis. They participate in the transcriptional modulation of gene expression levels via binding to *cis*-elements in gene promoters. To control anthocyanin biosynthesis, the EsMYB90 transcription factor from *Eutrema salsugineum* upregulates *CHS* and *CHI* gene expression and the activities of phenylalanine ammonia-lyase (PAL), ANS, UFGT, and DFR enzymes, and promotes the flowering of 35S: EsMYB90 tobacco transgenic plants [29]. SmbHLH13 binds to and upregulates the structural genes *SmCHS* and *SmF3H*, thereby promoting anthocyanin biosynthesis [30]. Ectopic expression of *CsWD40* isolated from green tea markedly elevated petal anthocyanin levels in transgenic tobacco [31].

The molecular mechanisms underlying anthocyanin biosynthesis in *D. candidum* remain largely unclear. We previously identified DcTT8, a bHLH transcription factor that combines with structural gene promoters of *D. candidum* and modulates anthocyanin biosynthesis [22]. AtMYB56, a transcription factor, responds to sucrose and plays a role in regulating anthocyanin accumulation in *A. thaliana* [32]. Additionally, PnMYB6 is clustered with AtMYB56, and the expression of *PnMYB6* is increased, which in turn triggers the expression of anthocyanin biosynthesis genes in *Phyllostachys nigra* [33]. However, the *DcMYB56* gene in *D. candidum* has not been studied. The red stems of *D. candidum* were first used to study the relationship between the transcription levels of *DcMYB56* and anthocyanin accumulation in *D. candidum*. The anthocyanin composition and concentrations in both *D. candidum* red and green stems were determined via ultra-performance liquid chromatography–tandem mass spectrometry (UPLC-MS/MS). Our findings revealed that the red coloration in stems was dependent on anthocyanin cyanidin derivatives. Moreover, the expression levels of the *DcCHS8*, *DcF3′H*, *DcF3′5′H*, *DcDFR1*, and *DcUFGT* genes in red stems increased relative to those in green stems, confirming the findings obtained for anthocyanin distribution in *D. candidum*. Yeast one-hybrid (Y1H) and transcription factor activity assays revealed that by binding to an extended region of the promoters of the *DcCHS8*, *DcF3′H*, *DcF3′5′H*, and *DcANS1* genes, DcMYB56 could regulate their expression levels, consequently regulating anthocyanin biosynthesis in *D. candidum*. Moreover, the anthocyanin content considerably increased in response to DcMYB56 overexpression in *Arabidopsis thaliana*. These findings shed valuable light on molecular anthocyanin synthesis in *D. candidum*.

## 2. Materials and Methods

### 2.1. Plant Materials and Growth Conditions

Red *D. candidum* (TaiHu-1) and green *D. candidum* (MingHu-1) are different varieties and have distinctly different appearances. The stems of red *D. candidum* are purplish red, whereas the stems of green *D. candidum* are green. The *D. candidum* varieties TaiHu-1 and MingHu-1 were obtained from Taining County and Shaxian County, Fujian Province, China, respectively. Two varieties of tissues were cultured on pine bark at 24 ± 2 °C in a greenhouse located at the Institute of Medicinal Plants, Sanming Academy of Agricultural Sciences, Sanming, Fujian, China. The wild-type (WT) and transgenic (T2) *A. thaliana* (ecotype Columbia) plants were subjected to growth on half-strength Murashige and Skoog media supplemented with a mixture of perlite/vermiculite/peat at a volumetric ratio of 1:1:1, while *Nicotiana benthamiana* plants were cultivated at a volumetric ratio of perlite/vermiculite/peat soil of 1:1:1 in a greenhouse. The conditions in the greenhouse were 22 °C, relative humidity of 40~60%, and a 16 h/8 h light/dark photoperiod.

### 2.2. Anthocyanin Composition and Contents

The total anthocyanin levels of *D. candidum* stems were determined via a pH differential approach. One g of stem sample was introduced into a mixture of methanol and 0.05% (*v*/*v*) HCl (5 mL) and stored for 12 h in the dark. The supernatants were then transferred to a 50 mL test tube. These steps were performed twice to ensure the complete extraction of the anthocyanins. The solution absorbance was detected at wavelengths of 510 and 700 nm with a UV-2550 spectrophotometer (Shimadzu Corporation, Kyoto, Japan). The anthocyanin level per g fresh weight of stem was computed as follows: ((A530-A657)/mg tissue fresh weight) × 1000. The red and green stem samples of *D. candidum* were pulverized with liquid nitrogen, and each sample (0.1 g) was subsequently introduced to a 4 mL solution that contained 70% (*v*/*v*) methanol and 0.1% (*v*/*v*) formic acid. The suspension was vortexed for 1 min and then ultrasonicated for 30 min at 40 Hz and 20 °C, followed by 10 min of centrifugation (10,000× *g*). Finally, it was injected into a benzene hexyl column (2.1 × 150 mm and 3.5 μm) in a Shimadzu 30A UPLC (Shimadzu Corporation, Kyoto, Japan) at 40 °C with an injection volume and column flow rate of 30 μL and 0.25 mL·min^−1^, respectively. Mobile phase A included 1% (*v*/*v*) formic acid and 5% (*v*/*v*) methanol, whereas mobile phase B included 1% (*v*/*v*) formic acid with methanol, which was selected on the basis of the properties of the anthocyanin monomers. The gradient elution process included 0–65% B over 0–3.1 min, 65% B over 3.1–8.0 min, 65–100% B over 8.0–8.5 min, 100% B over 8.5–12.5 min, 100–0% B over 12.5–13 min, and 0% B over 13–20 min. A SCIEX QTRAP 4500 triple quadrupole mass spectrometer (AB Sciex, Framingham, MA, USA) with Turbo V ion electrospray sources coupled to Analyst software v. 1.5 (AB Sciex, Framingham, MA, USA) was employed for the quantification of anthocyanins. The mass spectrometry conditions were as follows: positive ion mode, electrospray ionization, reaction monitoring, ion scanning range of 100–1000 *m*/*z*, pressure of the atomizer of 345 kPa, auxiliary gas flow rate of 10 L·min^−1^, collision gas medium, drying gas temperature of 350 °C, ion source temperature of 500 °C, ion spray voltage of 5500 V, and curtain gas pressure of 30 psi. The mass spectrometric parameters of the 18 anthocyanin monomers used for this study were previously described [34].

### 2.3. Quantitative RT-PCR (qRT-PCR)

Total RNA was extracted from the stems of *D. candidum* and the leaves of *A. thaliana* via RNA isolation kits (RN53-EASYspin Plus, Aidlab, Beijing, China) following specific protocols. Thereafter, RNA (about 2.5 µg) was subsequently used for synthesizing first-strand cDNA with a cDNA synthesis kit (SuperScript™IV First-Strand Synthesis System, Thermo Fisher Scientific, Baltics, UAB) as the template for qRT-PCR. For the quantitative analysis of the relative transcript levels, amplification was performed with a 7500 Fast Real-Time PCR system (Applied Biosystems, Foster City, CA, USA) and PowerUp SYBR Green Master Mix (Thermo Fisher Scientific, Austin, TX, USA). The 2^−ΔΔCT^ approach, which uses DcActin as a constitutive gene, was used for normalizing gene expression [35]. Each qRT-PCR was performed three times. The primers utilized for both RT-PCR and qRT-PCR are listed in Supplementary Table S1. 

### 2.4. Gene Cloning and Amino Acid Sequence Analysis

The DcMYB56 sequence was acquired from the RNA-Seq data of *D. candidum*. RT-PCR was adopted for cloning the full-length coding sequences of the genes. In addition, the BLAST search tool (https://blast.ncbi.nlm.nih.gov/Blast.cgi?PROGRAM=blastn&PAGE_TYPE=BlastSearch&LINK_LOC=blasthome) was applied to predict full-length protein sequences. The amino acid sequences were derived via the translation algorithm (https://web.expasy.org/translate/). The primers utilized in gene cloning can be obtained from Supplementary Table S2. 

The genomes of *D. candidum*, *O. sativa*, and *A. thaliana* were downloaded from the Genome Sequence Archive database (https://ftp.ncbi.nlm.nih.gov/genomes/all/GCF/001/605/985/GCF_001605985.2_ASM160598v2/, https://phytozome-next.jgi.doe.gov/info/Osativa_v7_0, and https://data.jgi.doe.gov/refinedownload/phytozome?organism=Athaliana&expanded=167, respectively), while the hidden Markov model (HMM) of the MYB binding domain (PF00249) was downloaded from the Pfam database (http://pfam.xfam.org/). The amino acid sequences containing the MYB domain were identified via HMMER software, version 3.3, with an e-value cutoff value of 0.01. The HMM search results were subsequently further verified using SMART, the Pfam website, and NCBI-CDD analyses.

The amino acid sequences of 374 *D. candidum* MYB, 267 *O. sativa* MYB, and 281 *A. thaliana* MYB genes of the three species were aligned via MAFFT software (version 7.475) (parameters: —localpair—maxiterate 1000—reorder), and then the ModerFinder software IQTREE (version 2.0.3) was used to determine the optimal model for constructing this phylogenetic tree. The model selection adopts JTT + R. The evolutionary tree was constructed via the maximum likelihood (ML) method, and the BootStrap parameter was set to 1000 repetitions, increasing the reliability and credibility of the branch results of the established tree. The final Newick-formatted evolutionary tree file was uploaded to the iTOL v.6 (https://itol.embl.de/) online tool for annotation and beautification.

### 2.5. Subcellular Localization Prediction

The fusion vector Pro-35S::DcMYB56 was first constructed and transfected into *Agrobacterium* strain GV3101. Thereafter, the DcMYB56 protein was subjected to a transient expression assay in leaf epidermal cells of *N. benthamiana* to predict DcMYB56 subcellular localization. For nuclear staining, we utilized 4′,6-diamidino-2-phenylindole (DAPI). GFP fluorescence signals inside agroinfiltrated *N. benthamiana* leaf samples were visualized via a confocal laser scanning microscope (Leica SP8 Microsystems, Wetzlar, Germany). Supplementary Table S2 displays all the primers used for vector construction.

### 2.6. Yeast One-Hybrid Assay

All DcMYB56 coding sequences were fused to the pGADT7 AD vector (Clontech Laboratories, Mountain View, CA, USA). Other vectors and optimal 3-amino-1,2,4-triazole (3-AT) contents used for this study have been previously described [25]. Y187 yeast strain cells (Clontech Laboratories) were then transfected with the constructed recombinant vectors, e.g., AD (AD-DcMYB56) and pHIS2 (pHIS2-Promoters). The growth of the yeast strain on the SD/-LTH media containing the optimal doses of 3-AT was monitored. All primers used for vector construction are presented in Supplementary Table S3.

### 2.7. Transcriptional Factor Activity Assay

Promoter activity analysis was performed via a transcriptional activity assay [36,37]. The binding of the coding sequence of DcMYB56 to 35S-LUC-GUS was induced via the promoter CaMV35S gene. The vectors and optimal concentrations of 3-AT were previously described [22]. The plasmids transformed into *Agrobacterium* strain GV3101 were cultivated on liquid yeast extract peptone (YEP) medium. Thereafter, the samples were centrifuged to obtain *Agrobacterium* GV3101 cells, which were suspended in infiltration buffer containing 100 µM acetosyringone, 10 mM MgCl_2_, and 10 mM MES at OD600 = 0.5. To analyze transcription factor activity, needleless syringes were used to infiltrate *Agrobacterium* GV3101 containing the recombinant plasmid into *N. benthamiana* leaf samples. Three days later, we determined the luciferase and GUS activities with the substrates luciferase and 4-methylumbelliferyl β-D-glucuronide, respectively. Every assay was performed at least five times via a microplate reader. All primers utilized in this work are listed in Supplementary Table S4.

### 2.8. Establishment of Transgenic A. thaliana Plants

The fusion vector Pro-35S::DcMYB56 was first constructed and transfected into *Agrobacterium* strain GV3101. The *A. thaliana* transformation used simple dipping of developing floral tissues into a solution containing *Agrobacterium tumefaciens*, 5% sucrose, and 500 microliters per liter of the surfactant Silwet L-77. The repeated application of Agrobacterium improved the transformation rates and overall yield of the transformants about twofold. The plants were covered for 1 d to retain humidity after inoculation. The validity of the glufosinate ammonium (Basta) selection method used to detect transformed progeny seedlings was determined.

### 2.9. Statistical Analysis

For the experiments, statistical analysis was performed via Student’s *t*-test. The asterisks indicate significant differences from three biological replicate experiments (* *p* <0.05, ** *p* <0.01, and *** *p* <0.001); GraphPad Prism v10.5.0 was used for statistical analysis.

## 3. Results

### 3.1. Cyanidin Derivatives of Anthocyanins Related to the Red Color of D. candidum Stems

**Figure 1:**
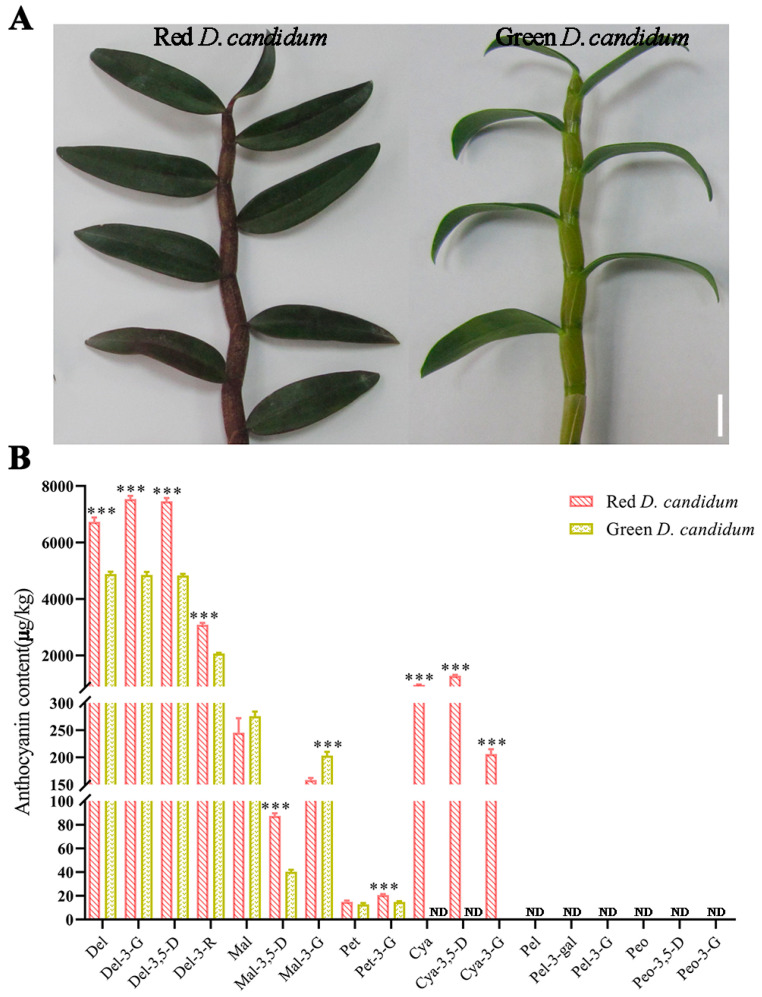
Anthocyanin contents in the two *Dendrobium candidum* phenotypes. (**A**) Left: *D. candidum* with red stems. Right: *D. candidum* with green stems. Bar = 2 cm. (**B**) Anthocyanins in red and green *D. candidum* determined using UPLC-MS/MS. Del, delphinidin; Del-3-G, delphinidin 3-*O*-β-D-glucoside; Del-3,5-D, delphinidin 3,5-diglucoside; Del-3-R, delphinidin 3-*O* rutinoside; Mal, malvidin; Mal-3,5-D, malvin; Mal-3-G, malvidin 3-galactoside; Pet, petunidin; Pet-3-G, petunidin 3-*O*-β-D-chloride; Cya, cyanidin; Cya-3,5-D, cyanidin; Cya-3-G, kuromanin; Pel-3-gal, pelargonidin; Pel, pelargonin; Pel-3-G, callistephin; Peo, peonidin; Peo-3,5-D, peonidin-3,5-diglucoside; Peo-3-G, peonidin 3-*O*-glucoside. The data are presented as the means ±SDs of three biological replicates. Statistical significance was analyzed using Student’s *t*-test (*** *p* < 0.001); GraphPad Prism was used for statistical analysis. “ND” represents “not detected.”

The TaiHu-1 stem was purple-red and rich in anthocyanin (Figure 1A, left) and the MingHu-1 stem was green (Figure 1A, right). The anthocyanin composition and contents in *D. candidum* stems were analyzed via UPLC-MS/MS to evaluate differences in coloration. These anthocyanins include delphinidin (Del), delphinidin 3-*O*-β-D-glucoside (Del-3-G), delphinidin 3,5-diglucoside (Del-3,5-D), delphinidin 3-*O*-rutinoside (Del-3-R), malvidin (Mal), malvin (Mal-3,5-D), malvidin 3-galactoside (Mal-3-G), petunidin (Pet), petunidin 3-*O*-β-D-glucoside chloride (Pet-3-G), cyanidin (Cya), cyanin (Cya-3,5-D), kuromanin (Cya-3-G), pelargonidin (Pel-3-gal), pelargonin (Pel), callistephin (Pel-3-G), peonidin (Peo), peonidin 3-*O*-glucoside (Peo-3-G), and peonidin-3,5-diglucoside (Peo-3,5-D). Del, Del-3,5-D, Del-3-R, Mal, Mal-3,5-D, Mal-3-G, Pet, Pet-3-G, Cya, Cya-3,5-D, and Cya-3-G were detected in *D. candidum* stems (Figure 1B). More anthocyanins were found in *D. candidum* red stems than in *D. candidum* green stems. Only red stems were found to contain Cya, Cya-3,5-D, and Cya-3-G. Moreover, except for Mal and Mal-3-G, the concentrations of all other anthocyanins in red stems were markedly greater than those in green stems. Despite having similar types of anthocyanins, there is a relative difference in the total contents of anthocyanins in different plant species, which also causes dramatic differences in plant color. The main colors of the flowers of crape myrtle trees are white, pink, and red-purple, owing to the presence of the main anthocyanins, such as delphinidin-3-*O*-glucoside, malvidin-3-*O*-glucoside, and petunidin-3-*O*-glucoside. The anthocyanin composition and level in the peels of sand pears are largely responsible for their red color [38]. Similar results were obtained for the flowers of *Dendrobium*-Phalaenopsis and tree peonies [39,40].

### 3.2. Isolation and Analysis of DcMYB56

**Figure 2:**
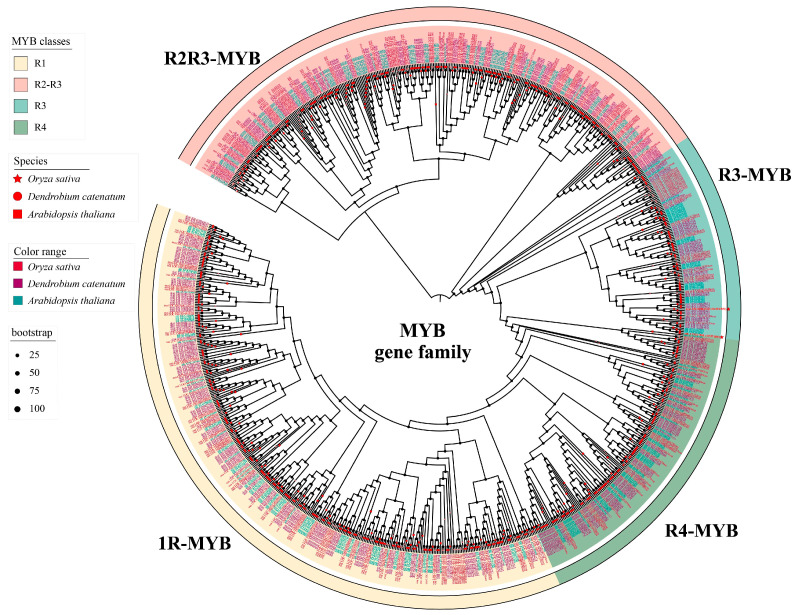
Phylogenetic relationships of *D. candidum*, *A. thaliana*, and *O. sativa* MYB proteins. The red circles represent the *D. candidum* MYB proteins. The red five-pointed stars represent the *O. sativa* MYB proteins. The red squares represent the *A. thaliana* MYB proteins.

The important roles of the orthologs of DcMYB56 isolated from *D. candidum* in anthocyanin biosynthesis have also been reported. DcMYB56 encodes 213 amino acids, which have a molecular weight and isoelectric point of 24.55 kDa and 9.86, respectively. To predict the functions of these TFs, phylogenetic trees were constructed. *A. thaliana* has 281 members, *D. candidum* has 374 members, and *O. sativa* has 267 members of the MYB family (Figure 2). Phylogenetic analysis indicated that DcMYB56 belongs to the R3-MYB subgroup of the clade and is clustered with AtMYB56. AtMYB56 is involved in anthocyanin biosynthesis [32]. Additionally, PnMYB6 is clustered with AtMYB56, and the expression of *PnMYB6* is increased, which in turn triggers the expression of anthocyanin biosynthesis genes in *Phyllostachys nigra* [33]. Therefore, we speculated that DcMYB56 was responsible for the regulation of anthocyanin biosynthesis.

### 3.3. The Expression and Nuclear Localization of DcMYB56 in Red and Green Stems of D. candidum

**Figure 3:**
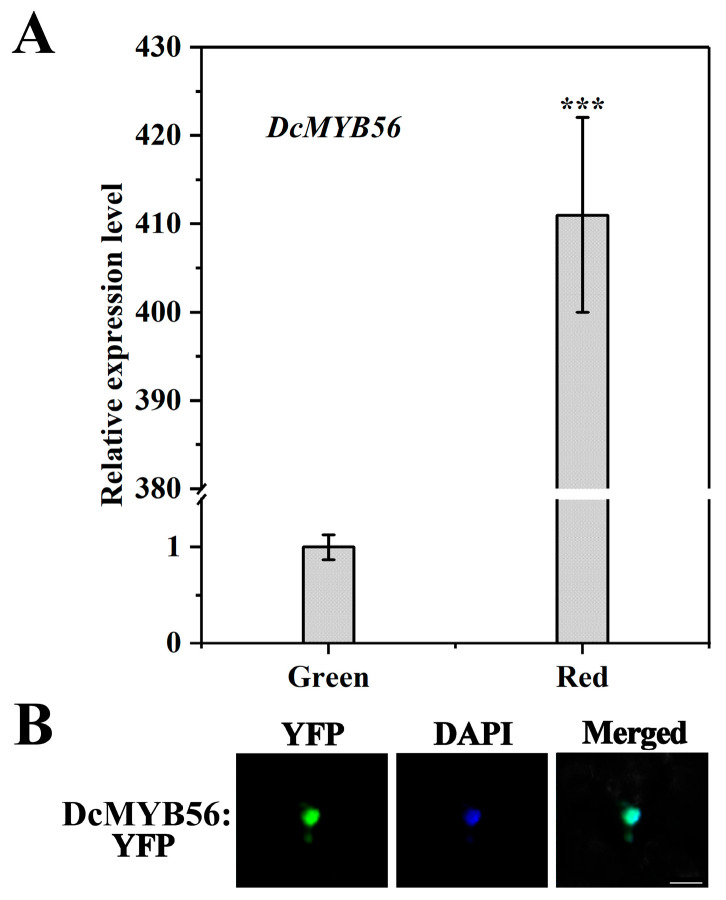
*DcMYB56* expression and subcellular localization. (**A**) Expression analysis of *DcMYB56* in red and green *Dendrobium candidum*. The data are the means of three biological replicates. Statistical significance was analyzed using Student’s *t*-test (*** *p* < 0.001), and GraphPad Prism software was used for statistical analysis. (**B**) Subcellular DcMYB56 localization in *Nicotiana benthamiana* leaf epidermal cells. Pro35s::DcMYB56-YFP localized to *N. benthamiana* leaf epidermal cell nuclei. 4,6′-Diamidino-2-phenylindole (DAPI) was used as a nuclear localization signal. The merged images show yellow fluorescent protein (YFP) and DAPI colocalization. Bar = 20 µm.

Transcriptomic analysis in previous studies revealed that *DcMYB56* (gene 21246) caused the upregulation of *D. candidum* red stems compared with their green counterparts [22]. To validate whether DcMYB56 is related to anthocyanin biosynthesis, qRT-PCR was performed. Compared with that in green stems, *DcMYB56* expression in *D. candidum* red stems markedly increased, with a relative difference in the expression level of about 410-fold (Figure 3A). Thus, DcMYB56 may be an important gene contributing to color differences in *D. candidum*. According to the results of fluorescence microscopy, the expression of the DcMYB56-YFP fusion protein in the pro35S::DcMYB56-YFP fusion expression vector in *N. benthamiana* leaves suggest the occurrence of DcMYB56-YFP nuclear localization (Figure 3B).

### 3.4. Expression of Key Anthocyanin Biosynthesis-Related Genes

**Figure 4:**
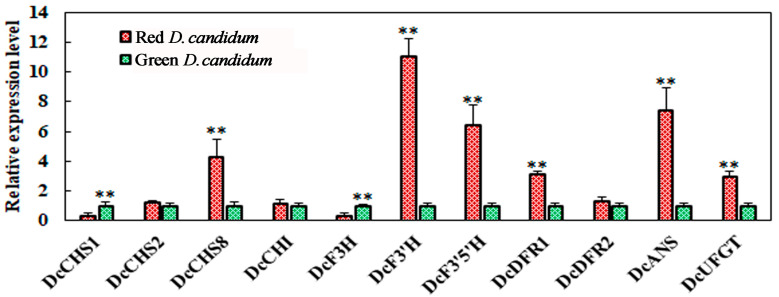
Relative anthocyanin biosynthesis-related gene expression levels. Transcriptional levels of 13 structural genes involved in anthocyanin biosynthesis in red and green *Dendrobium candidum* were analyzed using qRT-PCR. The gene expression levels were normalized to *DcACT* expression. The data are shown as the means ±SDs of three biological replicates. Statistical significance was analyzed using Student’s *t*-test (** *p* < 0.01), and GraphPad Prism software was used for statistical analysis.

To elucidate the molecular mechanisms associated with anthocyanin biosynthesis, the expression profiles of 11 associated genes, namely, *DcCHS1*, *DcCHS2*, *DcCHS8*, *DcCHI*, *DcF3H*, *DcF3′H*, *DcF3′5′H*, *DcDFR1*, *DcDFR2*, *DcANS*, and *DcUFGT,* were examined via qRT-PCR (Figure 4). Among them, *DcCHS8*, *DcF3′H*, *DcF3′5′H*, *DcDFR1*, and *DcUFGT* in red-stem *D. candidum* presented increased transcription levels compared with those in its green counterpart. In contrast, in green stems, *DcCHS1* and *DcF3H* transcription levels were greater than those in red stems.

### 3.5. DcMYB56 Regulates the Expression of Anthocyanin Biosynthesis-Related Genes

**Figure 5:**
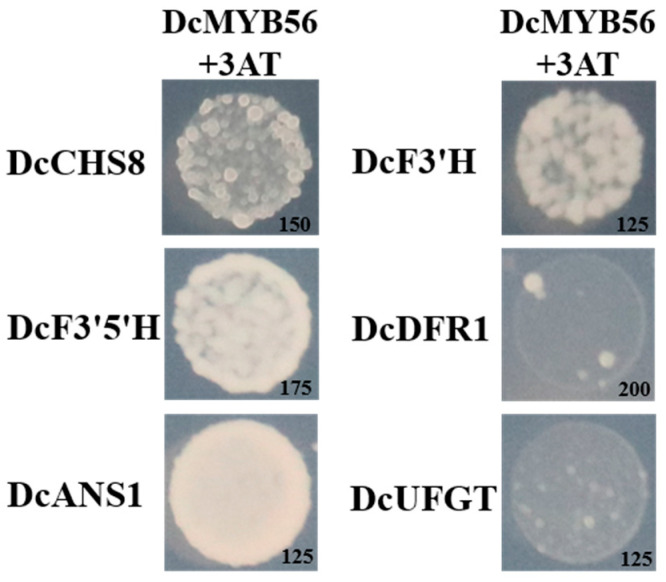
Yeast one-hybrid assays of the interactions of DcMYB56 with promoters of genes related to anthocyanin biosynthesis, including *DcCHS8*, *DcF3′H*, *DcF3′5′H*, *DcDFR1*, *DcANS*, and *DcUFGT*. The numbers in the lower-right corners of the images represent the optimal 3-amino-1,2,4-triazole (3-AT) concentrations. The results were obtained for three transformation experiments.

**Figure 6:**
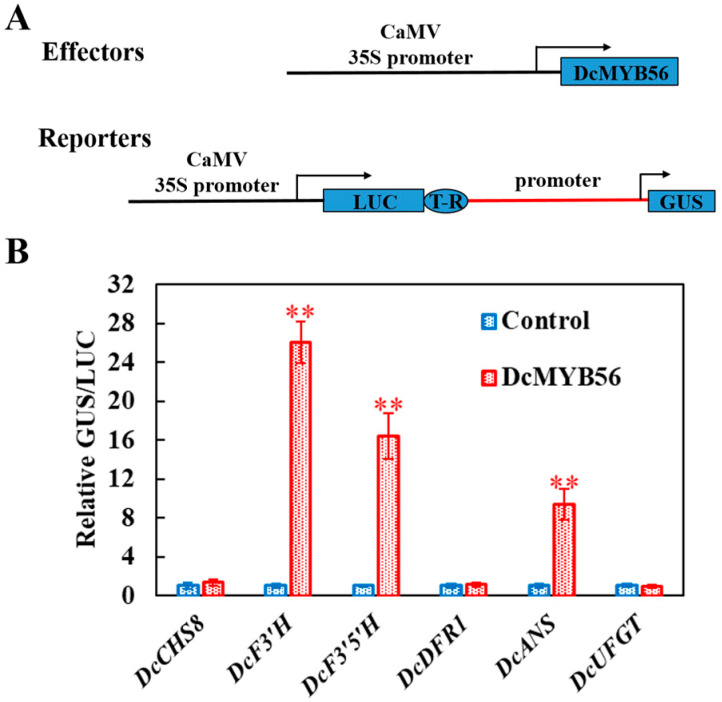
DcMYB56 regulates *DcF3′H*, *DcF3′5′H*, and *DcANS* expression. Activation of *DcCHS8*, *DcF3′H*, *DcF3′5′H*, *DcDFR1*, *DcANS*, and *DcUFGT* promoters by DcMYB56 analyzed via transient expression assays of effector and reporter plasmids in *N. benthamiana* leaves. (**A**) An effector reporter gene system was established to determine DcMYB56-mediated activation of six promoters of genes related to anthocyanin biosynthesis in *N. benthamiana* leaves. The effector vector contains DcMYB56 controlled by the CAMV35S promoter. The reporter vector contains the GUS reporter gene driven by the target gene promoter and LUC driven by CaMV35S for normalization. T-R, terminator; boxes, various DNA sequences. (**B**) Transcriptional activity of DcMYB56 toward six promoters analyzed via a reporter assay. The GUS:LUC activity ratio was 1 between the leaves transformed with the empty vector (control) and the target gene promoter. The data are the means and SEs of three biological replicates. Statistical significance was analyzed using Student’s *t*-test (** *p* < 0.01), and GraphPad Prism software was used for statistical analysis.

To assess whether DcMYB56 regulates gene expression, based on the RT-qPCR results, promoter fragments of six potential target genes were cloned from *D. candidum*. A Y1H experiment was carried out to analyze the potential binding of DcMYB56 to these promoters (Figure 5). The results of the Y1H experiment indicated that DcMYB56 interacted with the promoters of the *DcCHS8*, *DcF3′H*, *DcF3′5′H*, and *DcANS1* genes, leading to their activation, but did not interact with those of *DcDFR1* or *DcUFGT* (Figure 5). Transient transactivation assays were also performed to verify the relationships between DcMYB56 and target gene promoters related to anthocyanin biosynthesis (Figure 6A). β-glucuronidase (GUS) reporter gene activation by DcMYB56 triggered via the DcF3′H, *DcF3′5′H*, and *DcANS* promoters in *N. benthamiana* leaf samples was observed (Figure 6B). Thus, DcMYB56 may have induced *DcF3′H*, *DcF3′5′H*, and *DcANS* expression, which are related to anthocyanin biosynthesis, corroborating the Y1H assay results. Thus, DcMYB56 could modulate the expression of these three genes and, subsequently, anthocyanin biosynthesis.

### 3.6. DcMYB56 Overexpression Increases the Anthocyanin Content in A. thaliana

**Figure 7:**
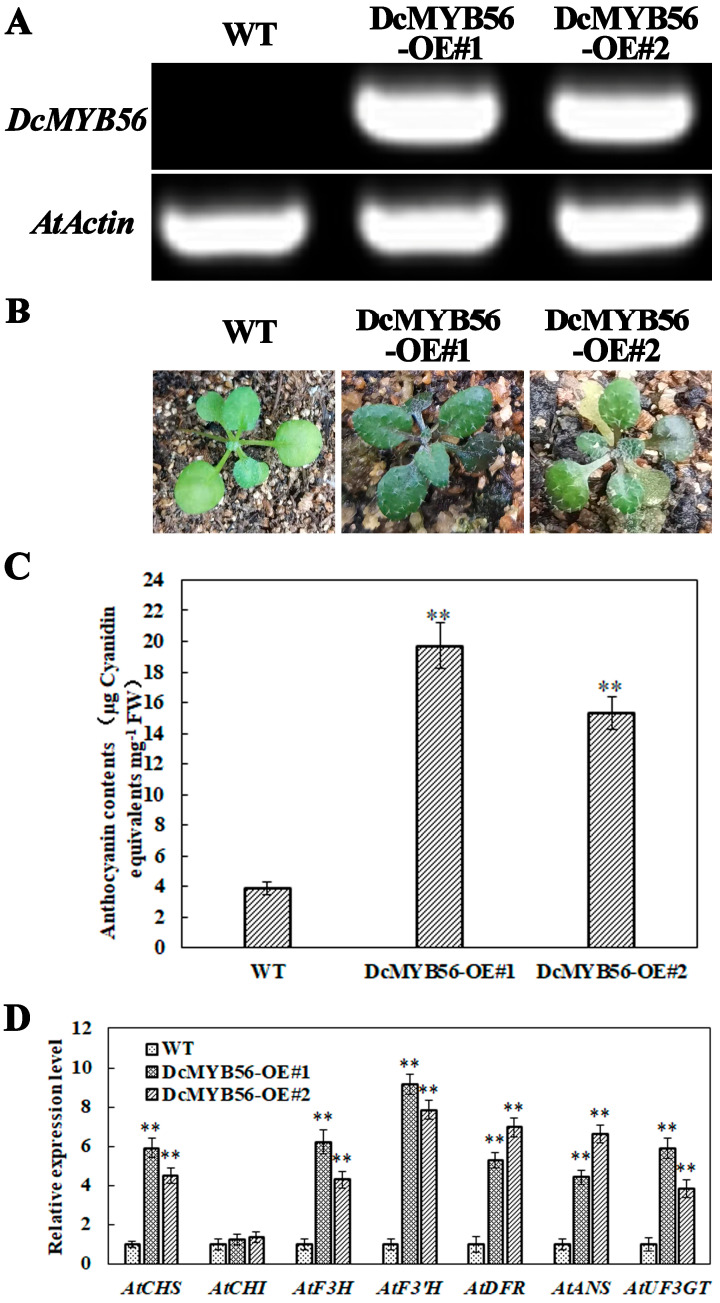
DcMYB56 overexpression increased the anthocyanin content in *A. thaliana*. (**A**) DcMYB56 overexpression in wild-type (WT) *A. thaliana* quantified via RT-PCR. AtActin was used as a housekeeping gene. (**B**) Twenty-day seedlings of WT *A. thaliana* (left), DcMYB56-OE#1 (DcMYB56 overexpression line 1, center), and DcMYB56-OE#2 (DcMYB56 overexpression line 2, right). Note that the DcMYB56-OE#1 and DcMYB56-OE#2 leaves were darker than those of the WT. (**C**) *Anthocyanin* content in the WT, DcMYB56-OE#1, and DcMYB56-OE#2 plants. The data are presented as the means and standard errors of three biological replicates. Statistical significance was analyzed via Student’s *t*-test (** *p* < 0.01), and GraphPad Prism software was used for statistical analysis. (**D**) Anthocyanin biosynthesis-related genes were upregulated in DcMYB56#1 and DcMYB56#2 plants compared with WT plants. WT gene expression levels = 1. AtActin was used as a housekeeping gene. The data are the means of three biological replicates for WT, DcMYB56-OE#1, and DcMYB56#2 plants. The error bars represent the standard errors of three biological replicates. Statistical significance was analyzed using Student’s *t*-test (** *p* < 0.01), and GraphPad Prism software was used for statistical analysis.

The production of transgenic *D. candidum* lines is both time-consuming and labor-intensive. A model plant, *A. thaliana*, was used to verify the role of DcMYB56 in the regulation of anthocyanin biosynthesis. The CaMV-35S promoter induced DcMYB56 overexpression in wild-type (WT) *A. thaliana* plants (Figure 7). Its expression and the anthocyanin content in the transgenic plants were then measured. OE 1 and OE 2 among a total of nine DcMYB56-overexpressing (OE) lines (DcMYB56-OE) obtained were examined. Substantial upregulation of DcMYB56 expression occurred in the OE lines, which also presented darker leaves and markedly greater anthocyanin contents than the WT plants did (Figure 7A–C, respectively). qRT-PCR was used to measure the transcript levels of anthocyanin biosynthesis-related genes in both the WT and the DcMYB56-OE plants. *AtCHS*, *AtF3H*, *AtF3′H*, *AtDFR*, *AtANS*, and *AtUF3GT* were upregulated in the latter (Figure 7D), indicating that DcMYB56 could positively regulate anthocyanin biosynthesis.

## 4. Discussion

Anthocyanins are key water-soluble red, purple, and blue pigments in plants [10]. They ubiquitously occur in flowers, stems, leaves, fruits, and other plant organs. Grape seeds, blueberries, purple cabbage, hawthorn bark, tea, and other plants are rich in anthocyanins [41,42]. Among them, the concentrations of Del, Del-3-G, Del-3,5-D, Del-3-R, Mal-3,5-D, Pet, Pet-3-G, Cya, Cya-3,5-D, and Cya-3-G in *D. candidum* red stems significantly increased compared with those in their green counterparts in the present study, with the last three detected only in the former, with high concentrations. The reason is that both the anthocyanin type and content directly or indirectly determine the plant color phenotype [43]. Delphinidin and its derivatives endow plant pigments with purple and blue colors, whereas cyanidin and its derivatives are responsible for producing red pigmentation [44]. Therefore, we propose that the main sources of red pigments in the stems of *D. candidum*, *Dendrobium officinale* [45], banana fruit [46], red *Camellia* flowers [47], and grape berries are Del, Del-3-G, Del-3,5-D, Del-3-R, Cya, Cya-3,5-D, and Cya-3-G [48]. These results suggest the role of delphinidin and cyanidin derivatives in conferring a purplish-red color to *D. candidum* stems.

Structural genes play crucial roles in anthocyanin biosynthesis. Compared with those in green *D. candidum* stems, structural genes in red stems were generally upregulated, which corresponds to the anthocyanin content determined in *D. candidum*. Cya-3,5-D synthesis in the anthocyanidin biosynthesis pathway is regulated by F3′H. Compared with green *D. candidum*, red *D. candidum* presented about a sevenfold increase in DcF3′H content. This difference may explain the absence of Cya, Cya-3,5-D, and Cya-3-G biosynthesis in the latter. Here, anthocyanin biosynthesis-related genes were upregulated, thereby increasing the anthocyanin concentration while increasing the color of the stem. The results of the Y1H experiment indicate that DcMYB56 interacted with the promoters of the *DcCHS8*, *DcF3′H*, *DcF3′5′H*, and *DcANS1* genes, leading to their activation, but did not interact with those of *DcDFR1* or *DcUFGT* (Figure 5). We suggest that the DcMYB56 transcription factor cannot bind to the promoters of the *DcDFR1* or *DcUFGT* genes in vitro. However, the increased expression levels of the *DcDFR1* and *DcUFG* genes in red stems of *D. candidum* may represent indirect regulation, which awaits further study. Our prior study revealed that *DcTTG1* expression is induced by light and that DcTTG1 binding to the promoters of *DcCHS2*, *DcCHI*, *DcF3H*, and *DcF3′H* can induce *DcTTG1* expression [49]. DcTT8 can bind the *DcF3′H* and *DcUFGT* promoters and finely regulate *DcF3′H* and *DcUFGT* expression [22]. The regulatory mechanisms of anthocyanin synthesis in different genetic materials of *D. candidum* also differ, and various types of transcription factors play different roles in different materials and environments.

MYB proteins are multifunctional transcription factors present in all eukaryotes. Their initially discovered MYB domain was a v-myb oncogene of avian myeloblastosis virus and, subsequently, the corresponding gene homolog c-Myb [50]. MYB transcription factors are crucial for regulating plant physiological processes such as secondary metabolism, growth, and stress response [51,52,53]. The strong conservation of MYB domains along the 1–4 amino acid sequence repeats (R motifs) enables MYB protein binding to DNA in plants. Each R motif contains about 52 amino acids, as well as three α-helices, with its third α-helix first recognizing DNA and then binding to its double helix structure. MYB precisely binds to the DNA sequence in this manner [54,55]. MYB transcription factors positively and negatively regulate anthocyanin biosynthesis. *MdMYB114* overexpression positively regulates anthocyanin biosynthesis and promotes anthocyanin accumulation in apple plants [56]. Through binding to the *CHS*, *ANS*, and *DFR* gene promoters and downregulating them, MaMYB4 negatively regulates anthocyanin biosynthesis in bananas [57].

MYB transcription factors play pivotal roles in modulating anthocyanin biosynthesis. Similarly, the regulation of anthocyanin biosynthesis by members of the MYB TF family, including SlMYB75, MdMYB1, RsMYB1, and MdMYB16, has been shown in different plant species [58,59,60]. According to the findings of the sequence alignment and phylogenetic tree in our study, the MYB transcription factor DcMYB56, which was isolated from *D. candidum,* was found to have a conserved domain in additional plants. The upregulation of DcMYB56 in red *D. candidum* in comparison with that in green *D. candidum* was demonstrated. Transcription factors can indirectly modulate anthocyanin biosynthesis through controlling structural gene transcription and expression [61]. The overexpression of *PSMYB57* in tree peonies resulted in the upregulation of *NtANS*, *NtCHS*, *NtDFR*, and *NtF3′H*, which caused further anthocyanin accumulation in tobacco [62]. MiMYB1 functions as an important regulatory factor for anthocyanin biosynthesis in mango fruit skin, which also accumulates anthocyanin in tobacco leaves [63]. *UFGT* was significantly downregulated with NtMYB2 overexpression in transgenic tobacco plants [64]. Among them, the most widely studied is the regulation of anthocyanin biosynthesis by the MYB-bHLH-WD40 (MBW) complex. R2R3 MYB TFs (PAP1, PAP2, MYB113, MYB114, or TT2) form MBW complexes with bHLH TFs (GL3, EGL3, or TT8) and the WD40 TF TTG1 [58]. In *Arabidopsis*, the MBW complex can directly bind to the promoters of *TTG2*, *TT8*, *F3′H*, *DFR*, *ANS*, *UGT79B1*, *UGT75C1*, *5MAT*, and *BLT* [65]. The TTG1-TT8/GL3-PAP1/PAP2/MYB113/MYB114 complex can activate *DFR* and *ANS* gene expression to affect anthocyanin biosynthesis [66,67]. There is no relevant research on the characteristic roles of MYB56 and the MYB56-dependent MBW complex in the regulation of anthocyanin biosynthetic structural gene expression. In future research, we will construct a yeast library of *D. candidum* and screen proteins that interact with DcMYB56 to explore the regulatory effect of the complex on anthocyanin synthesis. Based on our Y1H analysis results, DcMYB56 controls anthocyanin biosynthesis by modulating *DcF3′H*, *DcF3′5′H*, and *DcANS* gene expression. However, when DcMYB56 was overexpressed in *A. thaliana*, a significantly elevated content of anthocyanins was recorded, and the leaves turned red. Thus, DcMYB56 likely has a modulating effect on anthocyanin biosynthesis in *D. candidum*.

## 5. Conclusions

Our findings collectively provide empirical evidence for DcMYB56’s leading role in anthocyanin biosynthesis in *D. candidum*. DcMYB56 could combine with the promoters of genes associated with the regulation of anthocyanin biosynthesis, including *DcF3′H*, *DcF3′5′H*, and *DcANS*, and upregulate these genes. Consequently, DcMYB56 exhibited transcriptional regulation similar to that observed for its homologs in other plants, such as *Arabidopsis*. The findings of this study reveal the roles that MYB transcription factors play in governing anthocyanin biosynthesis and its associated mechanisms in *D. candidum*.

## Data Availability

Full-length proteins were predicted by BLAST search (https://blast.ncbi.nlm.nih.gov/Blast.cgi?PROGRAM=blastn&PAGE_TYPE=BlastSearch&LINK_LOC=blasthome). The amino acid sequences were derived with a translation algorithm (https://web.expasy.org/translate/). All data generated or analyzed during this study are included in this published article and its supplementary information files.

## Supplementary Materials

The following supporting information can be downloaded at: https://www.mdpi.com/article/10.3390/plants14121805/s1, Table S1: Primers used for qRT-PCR analysis. Table S2: Primers used for the subcellular localization analysis and overexpression in *Arabidopsis thaliana*. Table S3: Primers used for the yeast one-hybrid assay. Table S4: Primers used for the transcriptional activity assay.

## Abbreviations

qRT-PCR: quantitative real-time PCR; WT: wild type; GUS: β-glucuronidase; LUC: luciferase; MUG: 4-methylumbelliferyl-β-D-glucuronide; DAPI: 4′,6-diamidino-2-phenylindole; UPLC-MS/MS: ultra-performance liquid chromatography–tandem mass spectrometry; Del: delphinidin; Del-3-G: delphinidin 3-*O*-β-D-glucoside; Del-3,5-D: delphinidin 3,5-diglucoside; Del-3-R: delphinidin-3-*O*-rutinoside chloride; Mal: malvidin; Mal-3,5-D: malvin; Mal-3-G: malvidin 3-galactoside; Pet: petunidin; Pet-3-G: petunidin 3-*O*-β-D-glucoside chloride; Cya: cyanidin; Cya-3,5-D: cyanin; Cya-3-G: kuromanin; Pel-3-gal: pelargonidin; Pel: pelargonin; Pel-3-G: callistephin; Peo: peonidin; Peo-3,5-D: peonidin-3,5-diglucoside chloride; Peo-3-G: peonidin-3-*O*-glucoside; CHS: chalcone synthase; CHI: chalcone isomerase; DHK: dihydrokaempferol; F3H: flavanone 3-hydroxylase; PAL: phenylalanine ammonia-lyase; DFR: dihydroflavonol 4-reductase; DHQ: dihydroquercetin; F3′H: flavonoid 3′-hydroxylase; F3′5′H: flavonoid 3′,5′-hydroxylase; ANS: anthocyanin synthase; UFGT: UDP glucose: flavonoid-3-*O*-glucosyltransferase; and 3-AT: 3-amino-1,2,4-triazole.
