# Supplementary material for: The R3-MYB Transcription Factor DcMYB56 Regulates Anthocyanin Accumulation by Activating the Expression of Anthocyanin Biosynthesis-Related Genes in Dendrobium candidum
Source: Plants (Basel). 2025 Jun 12;14(12):1805. doi: 10.3390/plants14121805 (PMC12197011; doi:10.3390/plants14121805)
Supplement: Supplementary file 1 [file plants-14-01805-s001.zip › plants-3657393-supplementary.pdf]

Dendrobium candidum R3-MYB transcription factor DcMYB56 regulates anthocyanin accumulation by activating expression of the related genes

Ning Jia[1, #]*, Wei Ye[2, #], Jinlan Jiang[2], Peiyu Wang[2] and Jiqin Liu[3]*

[1] College of Agricultural and Forestry Science and Technology, Hebei North University, Zhangjiakou 075131, China

[2] Institute of Medicinal Plant Sciences, Sanming Academy of Agricultural Sciences, Shaxian 365050, China

[3] Institute of Plant Quarantine, Science and Technology Research Center of China Customs, Beijing 100026, China

* Correspondence: jianing0805@126.com (N.J.), 13811199324@163.com (J.L.)

# Co-first authors.

Supplementary Material

1 Supplementary Tables

Supplementary Table 1 Primers used for qRT-PCR analysis.

Primer name	Primer sequences (5'–3')
DcMYB56-8	GTA ATT CAA GTA ACG AGT GCA CCA GCT C
DcMYB56-9	AGT TGC TCC AAC ACC TAA GAA ATC AAT G
DcCHS1-5	GAT ACT CGA CCA AGT TGA AAT TAA GCT TGG
DcCHS1-6	GAA CGC TGC GTA GCA CAA CAG TTT CTA C
DcCHS2-5	CAT TCT AGA TCA GCT GGA CGA GAG AGT G
DcCHS2-6	GAA CAC TGC GGA GAA CGA CGG TAT C
DcCHS8-5	GGT TGA AGA AAA GCT TGG GCT AGA CG
DcCHS8-6	CTA ATT GGG CAC ACT GTG GAG C
DcCHI-5	CCC ACA GCT GTT GAT GAA TCT GCA TTA TC

DcCHI-6	GAT ACC TTC AAC GTA GGG GAA ACT GG
DcF3H-5	GTC TTT CGA TCG CGA CGT TCC AGA AC
DcF3H-6	CAT TTA AGA CCT TCG GCT TCG TTG CGG
DcF3'H-5	GCT TTC GAT CGC GAC GTT TCA GAA C
DcF3'H-6	CTA AGC ATT TTG TCC TGC TGC TCC
DcF3'5'H-5	GAA ATG ATT TCG AGC TCA TAC CGT TCG
DcF3'5'H-6	CTT CCG GCA AAC TCC AAT CAA AG
DcDFR1-5	CAA AGA CCC TGA GAA TGA AGT GAT ACA AC
DcDFR1-6	CGT ACG TAC CCA ACC GGT CAT CTT GAC
DcDFR2-5	GTG CGT AAG CTT CTC TTC AAA GAA GTT G
DcDFR2-6	TCA CTT AAC AGC AAT CTG TTC TTT ATT CTC CTC
DcANS-5	CGG ATT TCT TGG GCT GTT TTC TGC
DcANS-6	CTA CAC AAC CGC CTT GTC TCC GCT TC
DcUFGT-5	GAG AAG CTT TTG GTG GAG GTA TTG CAA ATT G
DcUFGT-6	CAA TTT CTC TAT CTC ATT ATG CGA TGA CCC
DcActin-1	TCC CAA GGC AAA CAG AGA AA
DcActin-2	GGC CAC TAG CAT ATA GGG AAA G
AtActin-1	CTC AAT CAT GAA GTG TGA TGT GG
AtActin-2	GAT CAA TTT TTA CCT GCT GGA ATG
AtCHS-1	GGC AAA GAA GCG GCA GTG AAG
AtCHS-2	CGG AAG GAC GGA GAC CAA GAA G
AtCHI-1	CTC TCT TAC GGT TGC GTT TTC G

AtCHI-2	CAC CGT TCT TCC CGA TGA TAG A
AtF3H-1	GAC CAA GTC GGT GGA TTA CAA GC
AtF3H-2	TCC TTC AAC AGG CTG AAC CG
AtF3'H-1	TTC CTT ACC TTC AGG CGG TTA TC
AtF3'H-2	CGA GAG TGG TGT TGG TGG ATG
AtDFR-1	CTT TGT TCG TGC CAC CGT TCG
AtDFR-2	TCC TTC CTC AGA TAA ATC AGC CTT CC
AtANS-1	GTT TGC AGC TTT TCT ACG AGG
AtANS-2	TGA GCA AAA GTC CGT GGA GG
AtUF3GT-1	TGT CAG ATC GTT TTG GTT CC
AtUF3GT-2	GAT TCT TCC TCA CTT TCT CAC

Supplementary Table 2 Primers used for the subcellular localization analysis and overexpression *Arabidopsis thaliana*.

Primer name	Primer sequences (5'–3')
DcMYB56-1	CCT ACT AGT ATG GAA ACT AGA GAG CTT CAT AAT GTG
DcMYB56-2	CCT ACG CGT AGT TGC TCC AAC ACC TAA GAA ATC AAT G
DcMYB56-3	CCT ACG CGT CTA AGT TGC TCC AAC ACC TAA GAA ATC AAT G

Supplementary Table 3 Primers used for the yeast one-hybrid assay.

Primer name	Primer sequences (5'–3')
DcMYB56-4	CCT CAT ATG GAA ACT AGA GAG CTT CAT AAT GTG
DcMYB56-5	CCT CTC GAG CTA AGT TGC TCC AAC ACC TAA GAA ATC AAT G

DcMYB56-6	CCT GTC GAC CTA AGT TGC TCC AAC ACC TAA GAA ATC AAT G

Supplementary Table 4 Primers used for the transcriptional activity assay.

Primer name	Primer sequences (5'–3')
DcMYB56-7	CCT GGT ACC ATG GAA ACT AGA GAG CTT CAT AAT GTG
DcMYB56-6	CCT GTC GAC CTA AGT TGC TCC AAC ACC TAA GAA ATC AAT G
